# Optimizing In Vivo Perfusion Assessment by Laser Doppler Flowmetry—Effects of Probe Geometry and Signal Normalization

**DOI:** 10.3390/diagnostics16071025

**Published:** 2026-03-29

**Authors:** Elisabete Silva, Marisa Nicolai, Luís Monteiro Rodrigues

**Affiliations:** CBIOS—Research Center for Biosciences & Health Technologies, Universidade Lusófona, Campo Grande 376, 1749-024 Lisboa, Portugal; p7425@ulusofona.pt (E.S.); marisa.nicolai@ulusofona.pt (M.N.)

**Keywords:** LDF data enhancement, perfusion normalization, probe geometry, reactive hyperemia, in vivo measurements

## Abstract

**Background/Objectives**: Laser Doppler flowmetry enables rapid and simple measurement of microcirculation. However, variations in probe configuration can influence signal acquisition, making it essential to understand each probe’s characteristics when selecting equipment for specific physiological assessments. Therefore, this study aimed to compare perfusion measurements obtained with single-fiber (VP1T) and multi-fiber (VP1T/7) probes and to evaluate the effects of normalization strategies. **Methods**: Nine healthy female volunteers were recruited. Probes were positioned on the palmar aspects of the index and middle fingers of both hands while participants underwent a standardized brachial artery occlusion protocol. Data are presented as mean ± standard error of the mean. Correlations were assessed using Pearson’s correlation coefficient. Coefficients of variation (CV) and intraclass correlation coefficients were calculated. Baseline normalization was applied to measurements. Statistical analyses were performed using Student’s *t*-test, with significance set at *p* < 0.05. **Results**: Analysis of the full protocol revealed significant positive correlations between probes, indicating consistent temporal perfusion patterns. The VP1T/7 probe yielded significantly higher perfusion values than the VP1T probe, although both exhibited similar CVs. Inter-probe reliability was good, and intra-probe reproducibility ranged from good to excellent, particularly for the VP1T/7 probe. During the reperfusion phase, significant differences were observed only for ipsilateral measurements obtained with the VP1T probe. Normalization effectively reduced variability, and significant differences during reperfusion were detected with both probes. **Conclusions**: Although the multi-fiber probe consistently recorded higher perfusion values, normalization was essential to reduce variability and to enhance the detection of microvascular reactivity parameters.

## 1. Introduction

Recent technological advances have provided more user-friendly, non-invasive instruments for assessing cardiac and hemodynamic indicators, including blood pressure and oxygen saturation, enabling widespread monitoring to promote cardiovascular health and prevent disease [1,2,3]. Cardiovascular diseases remain the leading global cause of death and disability [4], in part because current reliable markers fail to detect early circulatory homeostasis. Although the relationship between macro- and microcirculation is well established [5,6,7], the underlying adaptive mechanisms remain incompletely understood [5,8,9,10]. Together with greater mechanistic understanding, more reliable methods and indicators are needed to detect early markers of vascular dysfunction [5,6,7].

Significant innovations have supported this goal, including the development of state-of-the-art functional imaging systems (e.g., photoacoustic tomography), which are capable of quantification of perfusion with real-time structural visualization [11,12]. However, these tools remain very expensive and exploratory in many respects, resulting in limited use and cross-laboratory applicability [3,13,14]. More commonly used are ultrasound techniques to assess flow-mediated dilation in conduit arteries, or optical-based technologies such as photoplethysmography (PPG) or laser Doppler flowmetry (LDF), which quantify skin perfusion [9,15,16,17]. Related, Post-occlusive Reactive Hyperemia (PORH) is a very well-studied physiological maneuver applied to assess in vivo perfusion, typically measured using LDF, PPG, and laser speckle contrast imaging [18,19]. PORH refers to the transient increase in blood flow evoked after a brief period of arterial occlusion, allowing assessment of microcirculatory adaptation to that controlled hyperemia [6,18,19]. However, only a few studies have linked PORH alteration to cardiovascular disease [8,20,21]. Moreover, the application of this methodology to clinical practice is far from established, especially when compared with indicators such as blood pressure or electrocardiography [5,8,9,10]. On the one hand, the mechanisms involved in reactive hyperemia are not completely clear, which blurs and potentially compromises the interpretation of related outcomes [6]. Additionally, different laser systems imply different interactions of light with tissues, meaning that results from PPG, LDF, or other systems are not directly comparable [7,22]. LDF, regarded as a reference technology, detects blood cell movement via the Doppler shift of back-scattered laser light, generating a perfusion signal proportional to the quantity and velocity of the moving cells. LDF enables continuous, real-time monitoring of microvascular perfusion in the skin, showing high temporal resolution and generating a highly variable, pulsatile signal greatly dependent on the operator’s experience [7,23,24,25,26]. Therefore, substantial methodological heterogeneity remains even among LDF-based studies [27]. Moreover, available devices use different sampling frequencies, volumes, and depths, various fiber configurations, and are sometimes coupled to other sensors (e.g., temperature). All these factors influence the recorded signal amplitude, temporal dynamics, and reproducibility [6,9,18,19,23,28,29,30,31].

Moreover, measuring a physiological variable such as flow is inherently challenging because it arises from the integration of multiple biological processes operating on different timescales. Neural activity modulates vascular tone within milliseconds, whereas hormonal and metabolic regulators act over minutes to hours, and structural vascular adaptations may evolve over days or weeks. As a result, perfusion signals exhibit multiscale behavior, including marked variability, non-linearity, and sensitivity to local and systemic influences. These properties complicate the interpretation of absolute perfusion values and amplify the methodological heterogeneity already present between devices and probe configurations [3,7,12,18,32].

The present study evaluates how probe geometry and sampling volume influence the measured PORH response. The primary aims were (1) to determine whether a multi-fiber probe provides greater temporal stability and reproducibility of the PORH signal than a single-fiber probe, and (2) to test the impact of a normalization procedure to reduce inter-individual variability of the LDF signal and to improve comparability between probes. Ultimately, we expect to contribute to the broader effort of standardizing microcirculatory assessment, potentially expanding the use of PORH as a complementary biomarker in clinical research.

## 2. Materials and Methods

### 2.1. Participants

A convenience sample of nine female participants was recruited in the university community (Universidade Lusófona, Lisboa campus, Portugal, EU). All participants self-reported having no diagnosed cardiovascular, metabolic, or neurological disorders and were not taking regular medication or nutritional supplements. Anthropometric and hemodynamic characterization was performed at the beginning of the session, following standard recommendations for physiological research, including body mass index (BMI), mean arterial pressure (MAP), and ankle–brachial index (ABI) [33]. Characterization of the participants is depicted in Table 1. The only restriction imposed on participation was not to consume caffeine or alcohol or exercise up to 24 h before the experiments.

The study protocol was previously approved by the Ethics Committee of Universidade Lusófona Health School and conducted in accordance with the Declaration of Helsinki and its subsequent amendments for research involving human participants [34]. All individuals gave their written informed consent prior to the beginning of the study.

### 2.2. Experimental Procedure

Volunteers were required to respect a 15-min acclimation period, resting seated in a room with controlled temperature (22.0 °C) and humidity, before the beginning of the protocol.

A (blood) pressure cuff (Tensoval, Hartmann, Heidenheim, Germany) was placed on one arm, randomly chosen. Two types of single delivery probes were used—one with a single collecting fiber (VP1T) and the other with eight collecting fibers (VP1T/7) (both Moor Instruments, Cologne, Germany). The probes were placed on the palmar face of the index and the middle fingers, respectively, in both hands. Probes were connected to the MoorVMS-LDF system (Moor Inc., Axminster, Devon, UK). As the study was not designed to assess inter-individual variability, each volunteer contributed with up to 2 measurements, performed separately in time to minimize any influence of prior recordings. Additional measurements with randomized probe placement between the index and middle fingers were also performed.

The POHR protocol involved a 10-min initial registration (resting phase) followed by occlusion of the brachial artery for two minutes (below 200 mmHg) and a ten-minute recovery period. Skin blood perfusion was recorded with a sampling rate of 40 Hz. Measurements were performed by the same operators, two experienced investigators from our research lab.

### 2.3. LDF Recording and Data Analysis

Recordings were fully anonymized and data randomized. Perfusion was expressed in arbitrary perfusion units (PU) and acquired through the manufacturer’s software (Moor Instruments, Devon, UK).

Three analytical approaches of the absolute PU values were used. First, complete traces (22 min) of both hands were analyzed to determine the correlation between values recorded by VP1T and VP1T/7 probes. Second, the ten-minute initial perfusion recordings were used to determine mean perfusion values for each probe (VP1T and VP1T/7) and hand (ipsilateral and contralateral), and to calculate the coefficient of variation (CV) and intraclass correlation coefficients (ICC [2,1] and ICC [3,1]), allowing characterization of probes’ robustness under stable resting conditions. In this study, robustness was defined as temporal stability (assessed by baseline CV) and reproducibility (assessed by ICC [2,1] for inter-probe agreement and ICC [3,1] for intra-probe consistency). Signal-to-noise ratio was not calculated since the acquisition system provides processed perfusion units without access to raw Doppler frequency distributions. Third, mean perfusion values were determined for defined phases of the occlusion–reperfusion protocol—Phase I (30 s immediately preceding cuff inflation, used as baseline), Phase II (occlusion, 120 s), and Phase III (reperfusion), divided into early (first minute, capturing the hyperemic peak) and late (last three minutes of the protocol) segments. Additionally, temporal characteristics of perfusion were assessed in the ipsilateral arm by calculating the time to occlusion (TO) and the time to fast reperfusion (TR) for VP1T and VP1T/7 probes. TO was defined as the time interval between cuff inflation and the visually identifiable onset of the steep perfusion decrease, which corresponded to the rapid descending phase preceding the plateau of phase II. Similarly, TR was defined as the interval between cuff release and the onset of the steep ascending phase during reactive hyperemia (early phase III).

For comparison across probes and subjects, perfusion data obtained during the three above-mentioned phases, in both hands, were normalized to Phase I using the following formula:

Normalized perfusion = (value − mean Phase I PU)/mean Phase I PU.

### 2.4. Data Analysis and Statistics

When evaluating the full traces obtained with VP1T and VP1T/7 probes, the Pearson correlation was chosen due to the large sampling size per measurement (thousands of points recorded during each PORH maneuver). In such large datasets, minor deviations from normality have minimal impact on Pearson correlation (Central Limit Theorem).

Data are presented as mean ± SEM to represent the central tendency and the precision of the estimated mean for each phase. For comparisons between phases of the PORH protocol, normality of the differences (either between probes or between ipsilateral and contralateral limbs using the same probe) was tested using the Shapiro–Wilk test and visual inspection of Q-Q plots. No significant deviations from normality were detected, justifying the use of the paired Student’s *t*-test.

Temporal stability was assessed by calculating the CV over the ten-minute baseline period for each participant; lower CV values were interpreted as greater signal stability. Inter-probe reproducibility was evaluated using the intraclass correlation coefficient ICC [2,1], appropriate for assessing absolute agreement between different probes. Intra-probe consistency was evaluated using ICC [3,1], appropriate for assessing absolute agreement between different fingers measured with the same probe.

Statistical analysis of Pearson correlation and paired Student’s *t*-test was performed using GraphPad Prism version 8.0.1. CV and ICC values were calculated in Microsoft Excel using standard formulas. ICC values were interpreted according to Koo and Li [35]. An ICC value < 0.50 was considered poor, 0.50–0.75 moderate, 0.75–0.90 good and >0.90 excellent. A *p*-value < 0.05 was considered statistically significant.

## 3. Results

**Figure 1:**
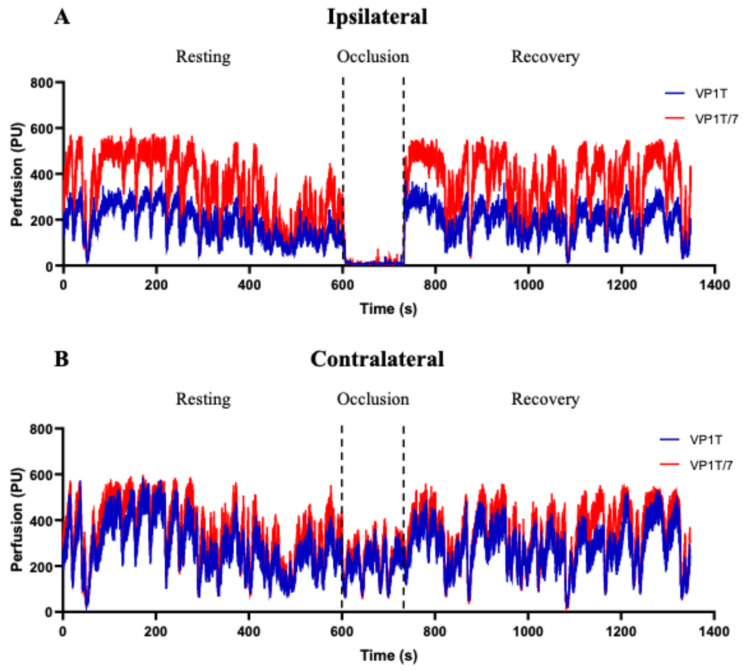
Representative traces of Laser Doppler Flowmetry (LDF), from a single participant. (**A**) Traces from the ipsilateral hand, where brachial occlusion was performed, and (**B**) traces from the contralateral hand. LDF signals were recorded using the VP1T (in blue) and VP1T/7 (in red) probes, placed on the index and the middle fingers, respectively. Values are expressed in perfusion units (PU).

As described, LDF recordings were obtained from both hands using the VP1T probes placed on the index fingers, while the VP1T/7 probes were placed on the middle fingers. Representative traces obtained with both probes, in the same volunteer, during the PORH protocol, are shown in Figure 1A,B.

Correlation analyses of the full PORH protocol (40 Hz sampling over 22 min) were performed to evaluate consistency between VP1T and VP1T/7 probes. Comparisons between the index and middle fingers (different probes) revealed positive, significant correlations in both the ipsilateral and contralateral hands, indicating consistent temporal dynamics across probes (Table 2).

Next, we used the ten-minute initial perfusion recordings to evaluate variability and consistency between probes and reproducibility across hands, using CV and ICC analyses. This period provides a good reference for assessing the probes’ performance, as it corresponds to a resting period. No differences were observed when comparing the mean perfusion values assessed with the same probes in opposite hands (222 ± 36 vs. 232 ± 45, *p* = 0.730 for VP1T probe in ipsi- and contralateral index fingers, respectively; 393 ± 44 vs. 307 ± 43, *p* = 0.532 for VP1T/7 probe in ipsi- and contralateral middle fingers, respectively). However, the mean values from the VP1T probes were significantly lower than those from the VP1T/7, both in the ipsilateral (222 ± 36 vs. 393 ± 44 PU; *p* = 0.006 for VP1T and VP1T/7, respectively) and the contralateral hand (232 ± 45 vs. 307 ± 43 PU; *p* = 0.037 for VP1T and VP1T/7, respectively), indicating a clear effect of probe geometry on absolute PU, despite the large inter-individual differences in perfusion. Interestingly, the CV values obtained for VP1T and VP1T/7 were similar, suggesting comparable relative dispersion of measurements despite differences in absolute PU. Results from the ICC analysis show that inter-probe reliability, VP1T versus VP1T/7, was moderate (ICC [2,1] = 0.71 for the ipsilateral hand, and =0.65 for the contralateral hand). Intra-probe reproducibility across hands was moderate for VT1P probes (ICC [3,1] = 0.61) and good for the VT1P/7 probes (ICC [3,1] = 0.90), indicating greater stability of the multi-fiber configuration when measurements were repeated in opposite limbs. Table 3 summarizes mean absolute perfusion values, CV, and ICC.

Additional measurements with randomized probe placement on index and middle fingers confirmed that basal perfusion differences between fingers are negligible and do not account for the observed differences between VP1T and VP1T/7 probes. Detailed data are provided in Supplementary Figure S1.

Following probe characterization, the analysis was extended to the occlusion–reperfusion maneuver to assess how the probes capture dynamic changes in microvascular perfusion. This approach allows direct comparison of performance under the PORH maneuver, including both the magnitude and temporal pattern of perfusion changes. To standardize comparisons and decrease the effect of natural fluctuations in the initial 10-min recordings (resting period), the 30-s segment immediately preceding cuff inflation (Phase I) was used as the reference for baseline perfusion.

**Figure 2:**
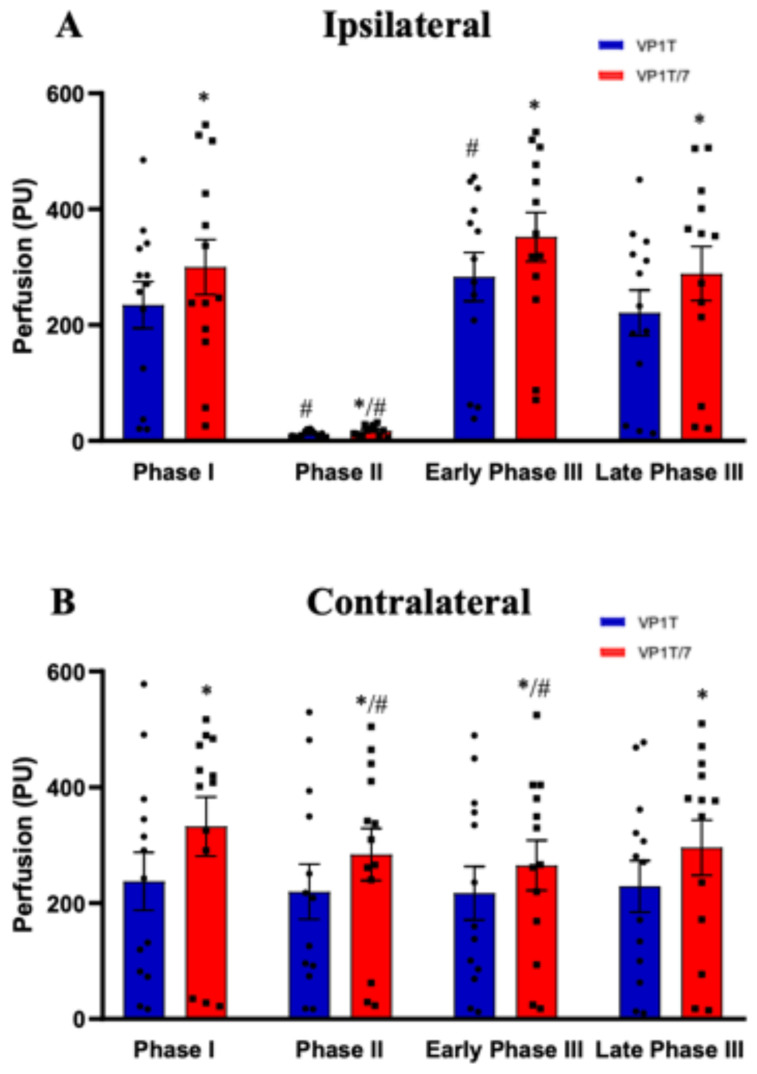
Absolute perfusion values during the post-occlusive reactive hyperemia (PORH) protocol. Bars represent absolute skin perfusion values (perfusion units, PU) measured by laser Doppler flowmetry during the PORH protocol in the ipsilateral (**A**) and contralateral (**B**) hands. Perfusion was recorded using the VP1T probe (in blue) and the VP1T/7 probe (in red). Data represent 13 independent measurements. Violin plots show perfusion values, median, and quartiles Data are shown for three predefined phases and represent 13 independent measurements. Bars show perfusion mean values with SEM. The asterisk symbol (*) indicates statistically significant differences between probes (VP1T/7 vs. VP1T) within the same phase, while the cardinal symbol (#) denotes statistically significant differences relative to Phase I (baseline) within the same probe.

In accordance with the date recorded during the ten-minute initial perfusion values, in this narrower time interval (Phase I—30 s before occlusion) the mean perfusion values from the VP1T probes were significantly lower than those from the VP1T/7, both in the ipsilateral (235 ± 40 vs. 300 ± 48 PU, mean values for VP1T and VP1T/7, respectively; *p* = 0.028) and contralateral hands (238 ± 50 vs. 333 ± 51 PU, mean values for VP1T and VP1T/7, respectively; *p* = 0.018) (Figure 2A,B).

No differences were observed when comparing the basal values from the index and middle fingers, using the same probe types. During occlusion (Phase II), a significant reduction in local perfusion was recorded with both probes in the ipsilateral hand (Figure 2A). Additionally, in Phase II and for the contralateral arm, significance was attained with VP1T/7 probes (Figure 2B). Zero perfusion was not recorded in the ipsilateral arm (Figure 1 and Figure 2).

Following cuff release, perfusion increased steeply in the ipsilateral hand and was significantly different from Phase II (Figure 2A). The maximum hyperemic response could be observed during the first minute of reperfusion (Figure 2A). The VP1T probe detected a significant increase relative to Phase I (235 ± 40 vs. 283 ± 42 PU; mean values for Phase I and early Phase III, respectively; *p* = 0.036). Despite the VP1T/7 probe having registered higher absolute values, it did not attain statistical significance in comparison to Phase I (300 ± 48 vs. 352 ± 42 PU, mean values for Phase I and early Phase III, respectively) (Figure 2A). Late Phase III, corresponding to the final three minutes of the protocol, showed that perfusion values in the ipsilateral hand had returned to levels that were similar to Phase I (Figure 2A).

**Figure 3:**
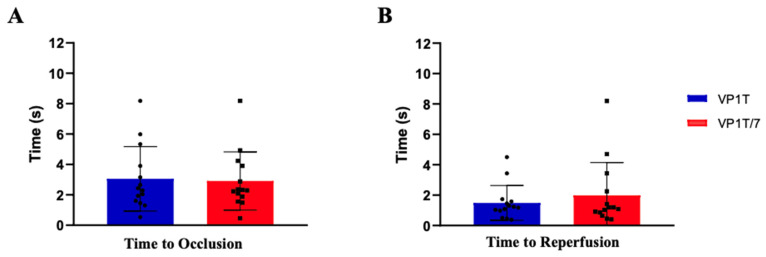
Temporal perfusion dynamics during arterial occlusion and reperfusion in the ipsilateral limb. Bar graphs illustrate the temporal parameters—(**A**) time to occlusion (TO) and (**B**) time to reperfusion (TR). (TR and TO were calculated using non-normalized perfusion unit (PU) values, directly derived from the LDF signals as mentioned in the materials and methods section. Bars represent mean ± standard error of the mean (SEM). A total of 13 independent measurements is shown. No statistically significant differences were observed between probes for either TO or TR.

To further characterize the dynamic response to occlusion and reperfusion, time to occlusion following cuff inflation and time to reperfusion after cuff release were calculated (Figure 3). No significant differences between probes were observed for the TO (3.05 ± 0.57 s vs. 2.91 ± 0.51 s, for VP1T and VP1T/7, respectively) or the TR (1.48 ± 0.31 s vs. 1.98 ± 0.58 s, for VP1T and VP1T/7, respectively).

**Figure 4:**
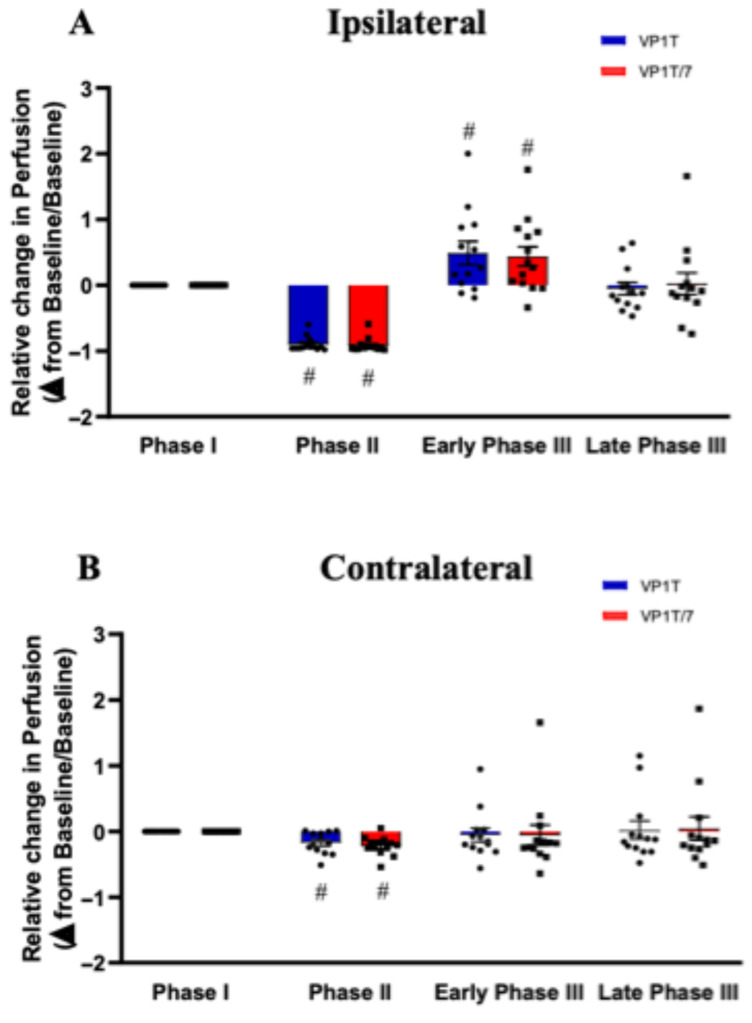
Normalized perfusion values during the post-occlusive reactive hyperemia (PORH) protocol. Bars represent normalized skin perfusion values obtained by laser Doppler flowmetry during the PORH protocol in the ipsilateral (**A**) and contralateral (**B**) hands. Perfusion was recorded using the VP1T probe (in blue) and the VP1T/7 probe (in red). Data are shown for three predefined phases and represent 13 independent measurements. Perfusion values were normalized to Phase I according to the formula mentioned in the Materials and Methods. Bars show perfusion mean values with SEM. The cardinal symbol (#) indicates statistically significant relative to Phase I (baseline) within the same probe.

Inter-individual variability was observed throughout the PORH protocol. These differences were less pronounced during Phase II in the ipsilateral hand, when perfusion reached its lowest values. Therefore, perfusion values were normalized for the mean values of Phase I. Following normalization, inter-individual differences were attenuated, and significance between groups increased (Figure 4).

In the ipsilateral arm, blood perfusion during early Phase III was significantly different from Phase I with both probes (from 0 in Phase I to 0.49 ± 0.17 and 0.41 ± 0.16 in the early Phase III, *p* = 0.010 and *p* = 0.015, for the VP1T and the VP1T/7 probes, respectively) (Figure 4A).

## 4. Discussion

This study compared two LDF probes, single delivery/single collecting fiber (VP1T) and a single delivery/eight collecting fibers (VP1T/7), using the same occlusion-reperfusion protocol, with the aim of identifying microvascular reactivity parameters that are independent of probe geometry and therefore potentially universal. The primary focus of this work was methodology, addressing how probe architecture influences perfusion data and the assessment of PORH, rather than the underlying biological mechanisms of microvascular regulation.

Measurements were assessed at the palmar fingertip, a glabrous skin region characterized by a dense and specialized microvascular network, organized into two main dermal plexuses: a superficial plexus just below the epidermis and an intermediate plexus deeper in the dermis, composed of interconnected capillary loops and arteriovenous anastomoses [2,7]. In these plexuses, there is a relatively uniform distribution of microvessels, which can make this region particularly suitable for reproducible microcirculation measurements. The palmar fingertip is supplied mainly by superficial dermal plexus. In this plexus, perfusion can be effectively assessed by LDF due to its optical penetration depth estimated between 0.6 and 1 mm [2,7]. Although LDF penetrates skin slightly deeper than PPG, measurements remain largely confined to the superficial plexuses, making this site suitable for reproducible assessment of microvascular perfusion [31]. In fact, despite the known high variability in absolute PU’s when sampling small areas, no significant differences were observed between the initial 10-min (non-normalized) perfusions obtained with the same probe type from corresponding fingers of distinct hands. According to these results, the fingertip appears particularly suitable for LDF measurements, providing absolute PU’s with lower site-to-site variability. This is in accordance with recently published work by Loktionova et al., also showing lower levels of microcirculatory variations in the fingers and the forehead skin, and between the right and left sides of the body [31].

In contrast, significant differences between the absolute perfusion obtained with distinct probe types persisted across all phases of the protocol in both hands. These results are likely due to distinct characteristics in probe geometry rather than biological heterogeneity. The VP1T and VP1T/7 probes have the same laser emission wavelength (785 nm ± 10 nm), which ensures similar optical penetration. However, probes differ in their receptor design. The VP1T/7 probe has a central delivery fiber surrounded by eight collecting fibers arranged in a 2 mm ring, whereas the single-fiber probe has a single collecting fiber. According to the manufacturer’s probe information catalogue, the multi-fiber configuration increases the effective sampling area and allows the probe to integrate perfusion from a broader portion of the dermal plexus, including regions that may contribute less to the signal in the single-fiber probe. As expected, the multi-fiber VP1T/7 consistently produced higher absolute readings when compared to the single-fiber VP1T. This outcome aligns with previous reports indicating that multi-fiber or multi-site probes enhance signal robustness and reduce spatial sampling error compared to single-point probes [6,10,36,37,38,39,40,41]. The larger sampling volume and possibly greater detection depth afforded by the multi-fiber configuration, due to the higher distance between the emitter and receptors, likely captures perfusion from a wider microvascular network, including capillaries at slightly greater depth or with heterogeneous distribution, that may be of value when performing perfusion measurements at less vascularized sites. Under the current experimental conditions, CV analyses showed similar relative variability between probes. The ICC analysis further supports the methodological robustness of LDF-derived perfusion measurements, indicating moderate inter-probe reliability (VP1T vs. VP1T/7) and good intra-probe reproducibility across hands for the VP1T/7, confirming this probe’s robustness.

In the VP1T/7 probe, signals from the eight fibers are averaged by the software, producing a higher baseline measurement. This methodological approach, aimed at increasing spatial averaging, likely attenuates the detection of rapid and localized perfusion changes. Phase-specific PORH analysis supports this interpretation: absolute perfusion values differed significantly between Phase I and early Phase III for the VP1T probe, whereas the corresponding comparison was not statistically significant for the VP1T/7 probe before normalization. However, the higher perfusion values obtained with the VP1T/7 probe may offer practical advantages in clinical and experimental settings where signal stability and spatial representativeness are prioritized over the detection of rapid dynamic changes. Measurements with the VP1T/7 probe may improve consistency in sites with heterogeneous or reduced perfusion, such as in peripheral arterial disease, diabetic microangiopathy, tissue viability assessment, or longitudinal follow-up studies requiring probe repositioning. Conversely, in protocols specifically designed to evaluate rapid dynamic vascular responses, such as early-phase PORH analysis, acute thermal stimulation, or pharmacologically induced transient hyperemic responses, the single-fiber VP1T probe may offer greater sensitivity to localized and rapid perfusion shifts.

The importance of normalization in PORH studies has been repeatedly highlighted [6,10,36,37,38,39,40,41]. Previous research has shown that expressing perfusion in relation to baseline reduces individual variability, stabilizes hyperemic curves, improves reproducibility of measurements, and enhances the detection of microvascular responses that might otherwise be less perceptible due to baseline differences or technical interference. Previous publications reported lower coefficients of variation for relative measures compared to absolute perfusion [10] and that normalized indices of hyperemia reduce inter-subject dispersion and produce more consistent reactive hyperemia curves [39,41]. Minson emphasized that normalization reduces momentary physiological variability such as cutaneous tone or temperature [38]. Additionally, Lefrandt et al. found better correlation between normalized hyperemic indices and clinical markers of microvascular function [37]. In the present study, normalization was essential not only to attenuate inter-individual variability (as previously described) but also to minimize variability attributable to probe geometry. Furthermore, the responses observed in the contralateral limb, consistent with previous reports of systemic neurovascular responses to unilateral maneuvers [40,42,43,44,45], became more clearly detectable after normalization.

As expected, this study confirms that normalization reduces inter-individual variability. Furthermore, it demonstrates that, when comparing probes with inherently different baseline signals, normalization becomes a necessary methodological step to ensure meaningful inter-probe comparison and accurate interpretation of PORH.

Importantly, in the ipsilateral hand, neither the TO nor the TR was significantly affected by the type of probe used. This suggests that temporal metrics of microvascular responses may be independent of absolute PU’s and probe geometry, providing reliable parameters for the evaluation of vascular adaptation, in line with previously published results [40,46,47].

Some limitations should be acknowledged. The sample size was small, and measurements were restricted to a single anatomical site. However, these choices were intentional and consistent with the exploratory, methodological aim of comparing probe performance and evaluating normalization strategies under controlled conditions. Therefore, in our opinion, these findings provide useful methodological insights to power laser Doppler perfusion studies.

Overall, the predefined aims of this study were achieved. The multi-fiber VP1T/7 probe demonstrated greater spatial reproducibility under stable baseline conditions, as reflected by higher ICC values and normalization of perfusion units to a short pre-occlusion reference interval, which reduced inter-individual and inter-probe variability, improving comparison between probes and enhancing the detection of phase-specific differences during PORH.

## 5. Conclusions

The VP1T/7 probe demonstrated greater robustness in terms of spatial reproducibility, while the VP1T probe showed greater sensitivity to rapid dynamic changes. Normalization of perfusion values to a short pre-occlusion reference interval reduced inter-individual and inter-probe variability. Subsequent analysis of the phases of PORH on these normalized data may provide more rigorous indicators that better describe cardiovascular adaptive physiology.

## Figures and Tables

**Table 1:** Characterization of the volunteers participating in the study (*n* = 9). Anthropometric and hemodynamic characteristics are presented as mean ± standard deviation of the mean (SEM).

	Mean ± SEM
Age (y)	41 ± 5
Body mass (kg)	64.4 ± 5.0
Height (m)	1.64 ± 0.01
BMI (kg/m^2^)	24.0 ± 1.8
MAP (mmHg)	91.3 ± 3.5
ABI	1.15 ± 0.04

**Table 2:** Correlation between VP1T and VP1T/7 traces obtained during PORH protocol. Mean r ± standard error of the mean (SEM) of individual Pearson correlations.

Hand	Mean r ± SEM	Range	*n*
Ipsilateral	0.83 ± 0.04	0.42–0.96	13
Contralateral	0.77 ± 0.05	0.41–0.94	13

**Table 3:** Baseline perfusion and reliability of laser Doppler flowmetry probes. Values expressed as mean ± standard error of the mean (SEM), *n* = 13.

Hand	Probe	Finger	Perfusion ± SEM (PU)	CV ± SEM (%)	ICC [2,1] [95% CI]	ICC [3,1] [95% CI]	*p*-Value VPT1 vs. VPT1/7		*p*-Value ipsi vs. Contra Same Probe	
							Perf	CV	Perf	CV
Ipsilateral	VPT1	Index	222 ± 36	39 ± 6	0.71	0.61	0.006 *	0.868	0.730	0.150
Ipsilateral	VPT1/7	Middle	393 ± 44	39 ± 8	-	0.96	-	-	0.532	0.972
Contralateral	VPT1	Index	232 ± 45	33 ± 6	0.65	-	0.037 *	0.194	-	-
Contralateral	VPT1/7	Middle	307 ± 43	39 ± 7	-	-	-	-	-	-

Perfusion units (PU); coefficient of variation (CV); intraclass correlation coefficients (ICC); Confidence interval (CI); * *p* < 0.05.

## Data Availability

Data available upon request to the corresponding author.

## Supplementary Materials

The following supporting information can be downloaded at: https://www.mdpi.com/article/10.3390/diagnostics16071025/s1, Figure S1: Randomized basal perfusion measurements. Each point represents the mean of basal measurement (10 min) from one hand, with probe placement randomized between the index and middle fingers. Values are presented as mean ± SEM (*n* = 6).

## Abbreviations

The following abbreviations are used in this manuscript:

PPG	Photoplethysmography
LDF	Laser Doppler flowmetry
PORH	Post-occlusive Reactive Hyperemia
y	Years
kg	Kilogram
m	Meter
mmHg	Millimeter of mercury
BMI	Body mass index
MAP	Mean arterial pressure
ABI	Ankle–brachial index
ECTS	Escola de Ciências e Tecnologias da Saúde
PU	Perfusion units
CV	Coefficient of variation
ICC	Intraclass correlation coefficients
s	Seconds
TO	Time to occlusion
TR	Time to fast reperfusion
SEM	Standard error of the mean
