# Supplementary material for: Optimizing In Vivo Perfusion Assessment by Laser Doppler Flowmetry—Effects of Probe Geometry and Signal Normalization
Source: Diagnostics (Basel). 2026 Mar 29;16(7):1025. doi: 10.3390/diagnostics16071025 (PMC13073997; doi:10.3390/diagnostics16071025)
Supplement: Supplementary file 1 [file diagnostics-16-01025-s001.zip › diagnostics-4133328-supplementary.pptx]

## Slide 1
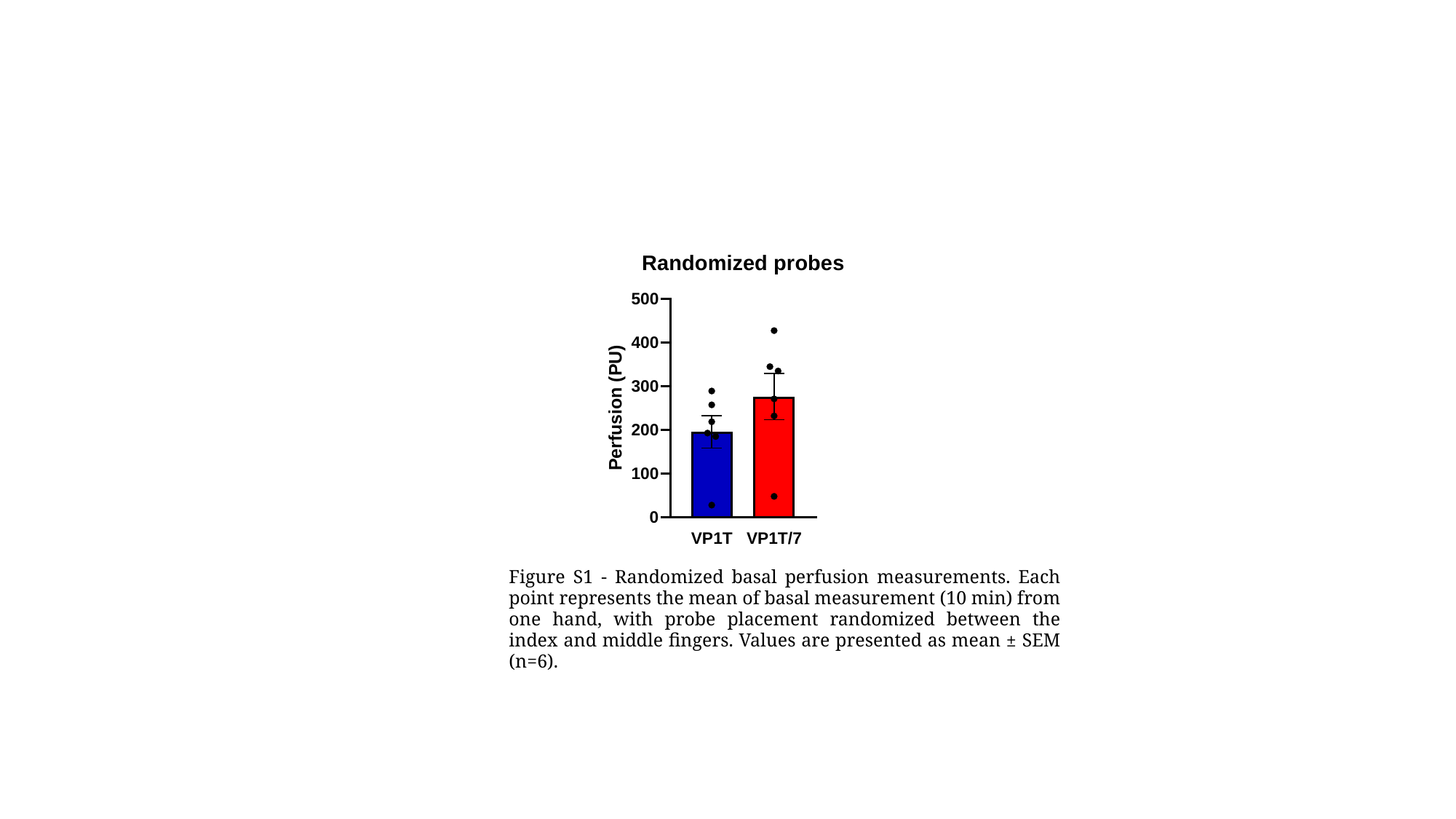

Figure S1 - Randomized basal perfusion measurements. Each point represents the mean of basal measurement (10 min) from one hand, with probe placement randomized between the index and middle fingers. Values are presented as mean ± SEM (n=6).
